# A prospective study of salvational intervention with ICS/LABA for reducing chronic obstructive pulmonary disease exacerbation under severe air pollution (SIRCAP) in Beijing: protocol of a multi-center randomized controlled trial

**DOI:** 10.1186/s12890-018-0771-9

**Published:** 2019-01-25

**Authors:** Tianyu Zhou, Yijue Zhong, Jiping Liao, Guangfa Wang, Xueying Li, Xiaosen Qian, Pingchao Xiang, Xinsheng Chen, Zhenyang Xu, Fengzhen Zhang, Xiaoguang Wang, Senlin Wang, Xiangxin Li, Chunxiao Yu, Yongxiang Zhang, Guoguang Xia, Li Dai

**Affiliations:** 1 Department of Respiratory and Critical Care Medicine, Peking University First Hospital, No.8 Xishiku Street, Xicheng District, Beijing, 100034 China; 2 Department of Medical Statistics, Peking University First Hospital, Beijing, China; 3 Department of Respiratory Medicine, Civil Aviation General Hospital, Beijing, China; 4 Department of Respiratory and Critical Care Medicine, Peking University Shougang hospital, Beijing, China; 5 Department of Respiratory Medicine, The Hospital of Shunyi District Beijing, Beijing, China; 6 Department of Respiratory and Critical Care Medicine, Beijing Luhe Hospital capital medical university, Beijing, China; 7 Department of Respiratory Medicine, Aerospace 731 Hospital, Beijing, China; 8 Department of Respiratory Medicine, Beijing Miyun Hospital, Beijing, China; 9 Department of Respiratory Medicine, Beijing Changping Hospital, Beijing, China; 10 Department of Respiratory Medicine, Beijing Jingmei Group General Hospital, Beijing, China; 11 Department of Respiratory Medicine, People’s Hospital of Beijing Daxing District, Beijing, China; 12 Department of Respiratory and Critical Care Medicine, Beijing Jishuitan Hospital, Beijing, China

**Keywords:** COPD, AECOPD, Air pollution, Budesonide/Formoterol, Salvational intervention

## Abstract

**Background:**

Chronic Obstructive Pulmonary Disease (COPD) is a major cause of morbidity and mortality all over the world. Acute exacerbation of COPD (AECOPD) not only accelerates the progression of disease, but also causes hospital administration and death events. Epidemiologic studies have shown air pollution is a high risk factor of AECOPD. However, there are rare technics or treatment strategies recommended to reduce severe air pollution related AECOPD.

**Methods:**

This is a multi-center, prospective, randomized and standard treatment parallel control clinical trial. Seven hundred sixty-four stable COPD patients in group B, C and D according to GOLD 2017 will be recruited and equally divided into two parallel groups, salvational intervention (SI group) and control group (CT group). Original treatments for participants include tiotropium (18μg once q.d), budesonide/formoterol (160μg/4.5μg once or twice b.i.d) or budesonide/formoterol (160μg/4.5μg once or twice b.i.d) with tiotropium (18μg once q.d). The savational intervention for SI group is routine treatment plus budesonide/formoterol (160μg/4.5μg once b.i.d) from the first day after severe air pollution (air quality index, AQI ≥200) to the third day after AQI < 200. CT group will maintain the original treatment. The intervention will last for 2 years. Primary outcome is the frequency of AECOPD per year and the secondary outcomes include the incidence of unplanned outpatient visits, emergency visits, hospitalization, medical cost and mortality associated with AECOPD per year.

**Discussion:**

The salvational intervention is a novel strategy for COPD management under severe air pollution. Results of the present study will provide reference information to guide clinical practice in reducing the air pollution related exacerbation of COPD.

**Trial registration:**

This study has been registered at www.ClinicalTrials.gov (registration identifier: NCT03083067) in 17 March, 2017.

## Background

Air pollution has become a serious public health issue in many regions, especially in China and other developing countries. Beijing is one of the most polluted cities in China, in which main pollutants are inhalable particulate matter (PM_10_) and fine particulate matter (PM_2.5_). PM_10_ is the particulate matter with a diameter of 10 μm or less and PM_2.5_ is with a diameter of 2.5 μm or less. Once inhaled, PM_2.5_ deposits in lung tissues and diffuses in blood causing airway and systematic inflammation [1]. A study on the 80,515 deaths (48,802 males, 31,713 females) recorded during 2004–08 in Beijing showed huge effects of air pollution on years of life lost. Every interquartile range (IQR) increase in PM_2.5_ and PM_10_ was associated with 15.8 and 15.8 years of life lost increase, respectively [2]. Despite the government has made efforts to control air pollution, this situation is extremely difficult to be fundamentally improved in short time.

Although preventable and curable, chronic obstructive pulmonary disease (COPD) is still a major cause of morbidity and mortality all over the world, and best characterized by persistent airway inflammation and progressive airflow limitation [3]. Acute exacerbation of COPD (AECOPD) not only accelerates the progression of disease, but also increases hospitalization and death events. What is worse, air pollution is proven to be a high risk factor of AECOPD [4–6], having a severe impact on patients’ quality of life and prognosis. Once inhaled, COPD patients reported to suffer from short breath and lung function decrease [7]. Meta-analysis evidence demonstrated that a 10 μg/m^3^ increase in PM_2.5_ was associated with a 2.36% (95% CI 1.00 to 3.73%) increase in the risk of COPD associated admissions [8]. A short-term (within 7 days) exposure of out-door air pollution rose the mortality rate of COPD by 1**,** 1 and 6% in China, the European Union and the United States respectively, and a long-term exposure increased the mortality rate by 10% [9]. In Beijing, with every increase of 55.89 μg/m^3^ of PM_10_, COPD related hospitalization and mortality would increase 29.69 and 46.35%, respectively [10]. Unfortunately, there are rare technics or treatment strategies recommended by any guideline around the world.

Thus, there is an urgent need for effective interventions to reduce AECOPD associated with severe air pollution exposure. Many issues must be taken into account: 1) geographic variation of air pollution, 2) measurement of air pollution exposure level, and 3) safety and effectiveness of the intervention.

In Beijing, air quality has been reported every hour automatically and summarized every 24 h since 2016 by Air Quality Automatic Monitoring System. In this system, there are 35 monitoring sites located in different districts of Beijing, and the monitoring area covers almost every corner in urban and main area in suburbs. Sulfur dioxide (SO_2_), nitrogen dioxide (NO_2_), carbon monoxide (CO), ozone (O_3_), inhalable particulate matter (PM_10_) and fine particulate matter (PM_2.5_) are monitored in each site. According to the 24-h mean concentration of PM_10_ and PM_2.5_, the air quality index (AQI) was calculated [11]. AQI of each site the day before is updated every single day with a diagrammatic sketch on official website of Beijing Municipal Environmental Protection Bureau [12]. Grades of AQI is also a guide to provide a health recommendation to the public (shown in Table 1). From Table 1, when AQI is above 200, in other words when it is under severe air pollution, individuals with respiratory diseases are not recommended to do outdoor activities. Our previous HEART study found that air pollution led to delayed inflammatory burst in lung lasting almost 3 days [6, 13]. After exposure to severe air pollution, airway inflammation of COPD patients get worsen [14]. At present, the pathophysiological process of exacerbation starts before patients have obvious symptoms. So it might be an optimal time for intervention from the first day to the third day after exposed to severe air pollution. Neither a pure treatment nor a simple prevention, this is a salvational intervention, aiming to rescue COPD patients from airway inflammatory burst caused by air pollution and to avoid AECOPD.

**Table 1:** Grading standard and health recommendation of AQI

AQI	AQI level	Concentration of primary pollutant (ug/m^3^)	Classification of AQI & representative color		Effect	Recommendation
0–50	Grade1	0–35	Excellent	Green	Almost no air pollution	All individuals can take normal activities
51–100	Grade2	35–75	Good	Yellow	Some pollutant have weak impact on some sensitive individuals	The sensitive individuals should reduce outdoor activities
101–150	Grade3	75–115	Slight pollution	Orange	Symptoms are aggravated in susceptible population; Irritative symptoms show in healthy individuals	Children, the elderly and people with cardiopulmonary diseases should reduce long-term and high-intensity outdoor exercise
151–200	Grade4	115–150	Moderate Pollution	red	Symptoms are further aggravated in susceptible population; Some influences on cardiopulmonary system are shown in healthy individuals	Children, the elderly and people with cardiopulmonary diseases should avoid l long-term and high-intensity outdoor exercise General population should moderately reduce outdoor activities
201–300	Grade5	150–250	Severe pollution	Purple	Symptoms are significantly aggravated in patients with cardiopulmonary diseases; Symptom are shown generally in healthy individuals	Children, the elderly, the people with heart disease or respiratory disease should avoid outdoor activities General population should reduce outdoor activities
> 300	Grade6	250–500	Extremely severe pollution	maroon	Tolerance of exercise decreased with obvious symptoms in healthy individuals; Some diseases appears in advance	Children, the elderly and patients should stay indoor General population should reduce outdoor activities

The information is original in Chinese on website< http://www.bjmemc.com.cn/g374/s1046/t1662.aspx > presented by Beijing Municipal Environmental Protection Bureau>

Inhaled corticosteroids (ICS) play an essential role on anti-inflammation, and beta_2_-agonists stimulate beta_2_-adrenergic receptors in order to relax airway smooth muscle. Combination of ICS and long-acting beta_2_-agonists (LABA) such as budesonide/formoterol and fluticasone/ salmeterol has effective synergistic action on improving symptoms, lung function, and life quality, as well as reducing exacerbation of COPD [15, 16]. Compared with fluticasone/ salmeterol, short-term treatment of budesonide/formoterol have a more rapid onset of action and improves pulmonary function and morning activities more greatly [17]. COPD patients treated with budesonide/formoterol have less AECOPD related emergency visits, hospitalization events and oral corticosteroids doses than those treated with fluticasone/salmeterol [18]. In the aspect of safety, budesonide/formoterol shows lower risk of pneumonia and pneumonia associated hospitalization and mortality [19]. Thus, budesonide/formoterol is chosen to be the ideal drug for the salvational intervention.

The salvational intervention for COPD patients under severe air pollution (AQI ≥ 200) is a short-term treatment with budesonide/formoterol (160μg/4.5μg once b.i.d) from the first day after the exposure to the third day after the exposure end. We hypothesize that it may be beneficial on reducing AECOPD frequency per year and AECOPD related visits, hospitalization, mortality, costs and so on.

## Methods

### Study design

This is a multi-center, prospective, randomized and standard treatment parallel control clinical trial. A total of 764 stable COPD patients diagnosed according to GOLD will be recruited and equally divided into two parallel groups, salvational intervention group (SI group) and control group (CT group). The Department of Respiratory and Critical Care Medicine in Peking University First Hospital is responsible for this research. Other 10 institutions participating in the study include Peking University Shougang Hospital, People’s Hospital of Beijing Daxing District, Beijing Jingmei Group General Hospital, Beijing Miyun Hospital, Beijing Changping Hospital, The Hospital of Shunyi District, Beijing Luhe Hospital capital medical university, Civil Aviation General Hospital, Beijing Jishuitan Hospital and Aerospace 731 Hospital. These 11 centers are located in the four corners and central of Beijing, covering area from urban to suburbs.

### Trial population

Investigators or physicians will explain the trial in detail to potential COPD participants from outpatient visits of each center. Total 764 eligible COPD patients will be enrolled after signing consent forms.

The inclusion criteria are 1) aged at 40–80 years old; 2) spirometry confirmed diagnose of COPD in group B, C and D according to GOLD 2017 with at least one acute exacerbation before and stable for at least 3 months; 3) quit smoking for more than 6 months; 4) be able to engage in daily activities; 5) have willing to participate in this study, follow the research program and have the ability to sign the informed consent; 6) Beijing residents; 7) can be contacted.

The exclusion criteria are 1) history of asthma, lung cancer, active pulmonary tuberculosis, bronchiectasis, diffuse lung disease (interstitial pneumonia, occupational lung disease, sarcoidosis et. al) and pleural disease; 2) history of lobectomy and / or lung transplantation; 3) predicted life expectancy less than 3 years; 4) history of severe psychiatric illnesses, mental disorders, neurological disorders, malignant tumors, chronic liver disease, heart failure, autoimmune diseases, chronic kidney disease; 5) never engage in outdoor activities; 6) plan to move out of Beijing in 3 years; 7) plan to carry out an indoor redecoration during the study; 8) alcoholism, drug abuse or abuse of toxic solvents; 9) allergic to the study drug or its ingredients, or have a clear contraindication of it; 10) participate in any other clinical trial; 11) cannot finish long term follow-up or be poor compliance; 12) do not provide consent.

### Baseline data collection

The information of demographic data, medical and medication history, smoking and exposure history, family history and main results of physical, laboratory, and lung function test (including bronchial dilation test) will be collected. Especially, region of daily activities like home or work address and the history of exacerbation of COPD in last 12 months will be recorded. Furthermore, such COPD assessment scales like the St. George’s Respiratory Questionnaire (SGRQ), COPD assessment test (CAT), Modified British Medical Research Council (mMRC) scale and BODE index will be noted.

### Washout period

Inhaled tiotropium (18μg once q.d), budesonide/formoterol (160μg/4.5μg once or twice b.i.d) or the combination of budesonide/formoterol (160μg/4.5μg once or twice b.i.d) and tiotropium (18μg once q.d) will be used as routine treatment. Inhaled medication will be withdrawn from all potential subjects prior to randomization (shown in Table 2).

**Table 2:** Inhaled pharmacological wash-out

Inhaled medication used before wash-out	Inhaled medication used during wash-out	Duration of wash-out
Tiotropium (18μg once q.d)	Maintain the original regimen	No washout, go directly into randomization
Budesonide/formoterol (160μg/4.5μg once or twice b.i.d)		
Tiotropium (18μg once q.d) + budesonide/formoterol (160μg/4.5μg once or twice b.i.d)		
No use of inhaled medication	Tiotropium (18μg once q.d)	Four weeks
Anticholinergic agents except tiotropium		
Inhaled corticosteroids only	Tiotropium (18μg once q.d) + budesonide/formoterol (160μg/4.5μg once or twice b.i.d)	
Inhaled β2-agonist only		
ICS/LABA except budesonide/formoterol		
LAMA+ICS/LABA except Tiotropium + budesonide/formoterol		

### Randomization

**Figure 1:**
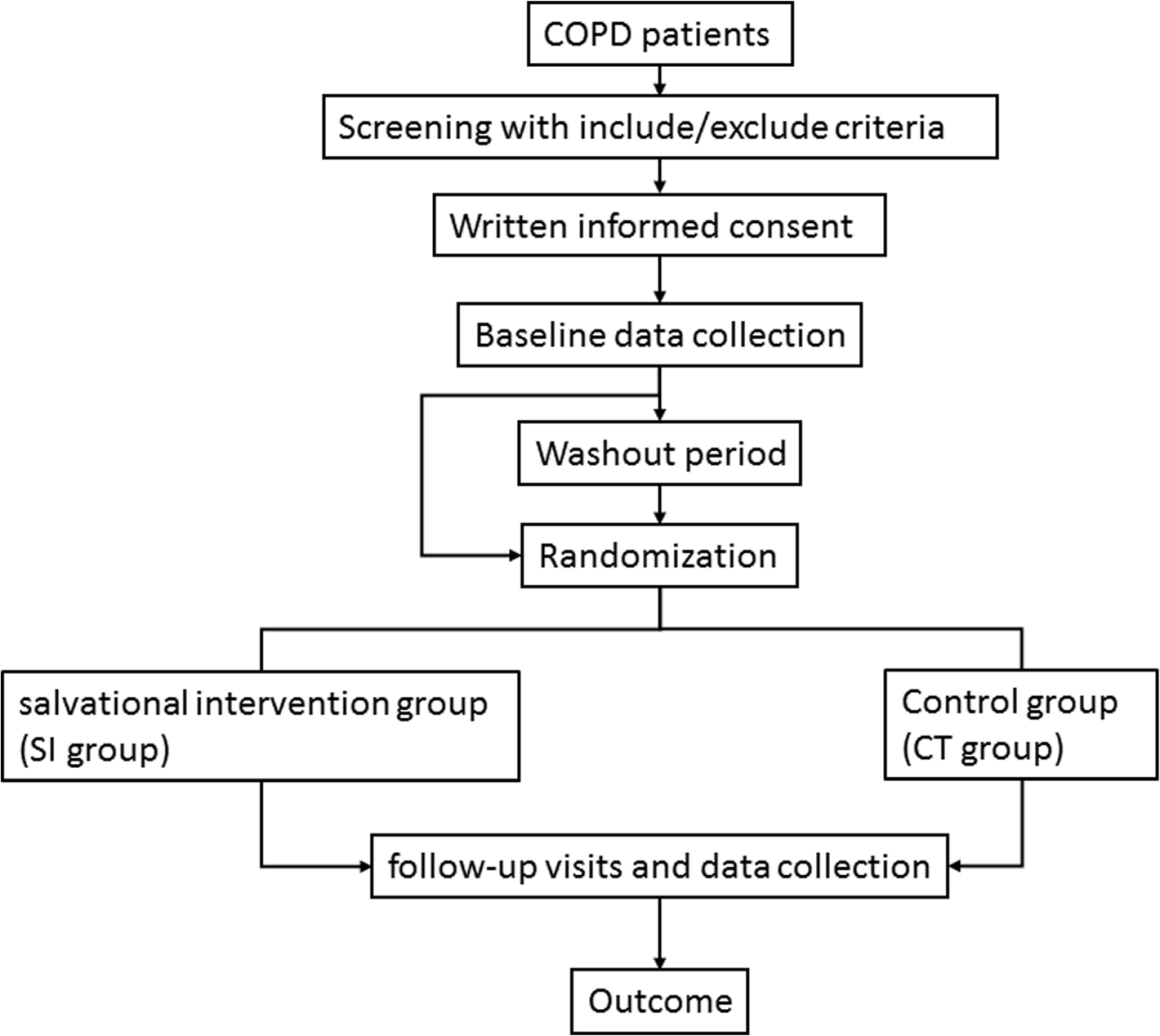
Flowchart of the study

The randomization will be stratified by centers and the random code will be designed in a 1:1 ratio (SI group or CT group), with a block size of 4, using the SAS 9.2 software package (SAS Institute, Cary, NC). The randomization results will be sealed in the envelopes until the end of the study. The flowchart of this study is shown in Fig. 1.

### Groups

After randomization, patients will be assigned to two groups, salvational intervention group (SI group) and control group (CT group). On the foundation of basic treatment strategies, inhaled budesonide/formoterol (160μg/4.5μg) will be used as an intervention drug for patients in SI group. When air quality index (AQI) is no less than 200, they will temporary add budesonide/formoterol (160μg/4.5μg once b.i.d) from the first day after exposure till the third day after AQI drops below 200. CT group maintains the original treatment. Intervention will be finished by telephone, message or E-mail. The intervention will last for 2 years. Daily AQI and whether the intervention is successfully accomplished during every intervention period will be recorded by investigators.

### Follow-up schedule

Besides baseline, patients will be formally visited 4 or 5 times during this trial: before randomization (for those who need pharmacological washout) and every 6 months after randomization. Questionnaires of SGRQ, CAT, mMRC scale and BODE index will be filled out and assessment of AECOPD will be collected. Meanwhile, information of physical, laboratory, and lung function test (including bronchial dilation test) will be collected, too. The details are listed in Table 3. Investigators are requested to contact subjects at least once a month by telephone, message or E-mail to ask patients about their respiratory symptoms and make a judgement about adverse events.

**Table 3:** Details of follow-up visits

Follow-up Visit	Baseline(V1)	Before randomize(V2)	The first time after randomization(V3)	The second time after randomization(V4)	The third time after randomization(V5)	The fourth time after randomization(V6)
	Screening period	4 weeks ± 1 day after washout period (in demand)	6 months ± 3 days after randomization	12 months ± 3 days after randomization	18 months ± 3 days after randomization	24 months ± 3 days after randomization
Informed consent	x					
Basic information	x					
Alteration of basic information		x	x	x	x	x
History of COPD	x					
Criteria	x					
Wash-out	x					
SGRQ	x	x	x	x	x	x
CAT	x	x	x	x	x	x
mMRC	x	x	x	x	x	x
BODE	x	x	x	x	x	x
AECOPD questionnaire	x	x	x	x	x	x
Physical examination	x	x	x	x	x	x
Lung function test	x	x	x	x	x	x
6 min walk time, 6MWT	x	x	x	x	x	x
Arterial blood gas analysis	x					x
Other medication	x	x	x	x	x	x
Adverse events report		x	x	x	x	x

What is more, all subjects have to complete diary cards during this study. Subjects are asked to record the daily dose of inhaled budesonide/formoterol (160μg/4.5μg) and score their status of cough, expectoration, dyspnea, physical activity and sleep on 5 points(from 0 = very well to 4 = very poor).

### Outcomes

Primary outcome is the frequency of AECOPD per year, which is defined as the incidence of AECOPD per patient per year.

Secondary outcomes include the number of unplanned outpatient visits, emergency visits, hospitalization, medical cost and mortality caused by AECOPD per year.

### Data collection and management

All baseline and follow-up data will be recorded on an online Case Report Form System. Project investigators of each center, study coordinators, investigators for intervention, investigators for follow-up visits and data managers will have accordingly authorized access to this system. Online data will be monitored carefully by two data managers every month. Complete online data will be exported into the EpiData3.10 database system.

### Data sharing

The study has no plan of data sharing.

### Adverse events

Adverse event is an unpredictable and infaust event that occurs during the study period, with or without the study intervention. Severe adverse event is an event that lead to death, threat of life, persistent disability or dysfunction, diagnose of tumor or other severe event during the study period. In our study, all drugs used for patients are routine medicine within recommended dosage. So our study will not produce additional risk on participants. However, adverse events may occur during daily use. All adverse events will be monitored carefully, managed promptly, and followed up until they are properly resolved, stabilized or recovered to normal. And occurrences of adverse events will be documented from the beginning to the end of the study including the time of occurrence, diagnosis, diagnosis time, management, duration of persistence, sequelae, and severity. The occurrences of severe adverse events will be reported to the Peking University First Hospital Institutional Review Board (IRB) as soon as possible. Severe adverse event will be analyzed every 6 months during the study. If there is a definite benefit (*P* < 0.01) or an obvious disadvantage(*P* ≤ 0.05), the research will be stopped after the discussion of all centers and the approval of the ethic committee.

### Management of AECOPD during study

AECOPD is a clinical diagnosis of an acute symptoms including increased cough, increased sputum volume and/or purulence, increased dyspnea, increased wheeze, chest tightness and fluid retention. When a participant suffers an acute exacerbation during this study, the salvational intervention will be suspended, and the participant will get treated and continue this study after 1 month of a stable condition. Symptoms, hospital admissions and costs associated with exacerbation will be recorded in next follow-up visit.

### Sample size

According to previous studies, every increase of 55.89 μg/m^3^ of PM_10_ increased COPD related hospitalization by 29.69% in Beijing, and a 10 μg/m^3^ increase in PM_2.5_ was associated with a 2.36% (95% CI 1.00 to 3.73%) increase in the risk of COPD associated admissions [8]. The Beijing Environmental Statement published in 2014 by Beijing Environmental Protection Agency showed monthly mean concentration grew from approximately 70 μg/m^3^ to 150 μg/m^3^ of PM_10_, and 52 μg/m^3^ to 150 μg/m^3^ of PM_2.5_ [20], suggesting an increase of at least 20% risk of exacerbation with the change of air pollution. Assuming that rate ratio of exacerbation frequency for SI group (salvational intervention group) is 0.85 compared with CT group (control group), a total of 610 subjects (305 for each group) are required to detect a 75% reduction of air pollution associated exacerbation at a 90% power with a two-sided significance level of 0.05. Considering a dropout rate of approximately 20%, a total of 764 participants will be enrolled in this study.

### Statistical analysis

Analysis will be performed on an intention-to-treat basis. All statistical analysis will be performed with SPSS 14.0 software (International Business Machines Corp., New York, USA). Two-tailed tests will be used in all statistical analysis, and *p* values < 0.05 will be considered to have statistical significance (unless otherwise specified).

A Poisson regression model will be used to calculate the rate ratio of frequency of exacerbation and the 95% confidence interval will be estimated. The influences associated with different centers or baseline data will be considered.

Numeric variables will be presented as mean (standard deviation) or median (minimum, maximum; or interquartile range) and categorical variables will be presented as number of cases (percentage). Accordingly, data will be analyzed with independent sample t test, Wilcoxon rank sum test, chi-square test, continuity correction Chi-squared test or Fisher’s exact test. Characteristics of baseline will be summarized with equilibrium test. Unplanned outpatient visits, emergency medical visits, hospitalization, medical cost and mortality caused by AECOPD per year will be compared between two groups.

## Discussion

This study points out an idea about salvational intervention for COPD patients under severe air pollution, which is a novel strategy of adding budesonide/formoterol (160μg/4.5μg) twice a day from the first day after severe air population (AQI ≥ 200) till the third day after the population end (AQI < 200). This multi-center, prospective, randomized and standard treatment parallel control clinical trial is aiming to figure out whether the salvational intervention will reduce the air pollution related exacerbation of COPD.

Airflow limitation, symptoms and exacerbation history are very important assessment for COPD pharmacological management. For patients with FEV1 < 80% of predicted value (after bronchodilator use), a history of exacerbations is a predictor of exacerbations, and exacerbations will become more frequent and more severe along with the progression of COPD [21]. GOLD 2017, presented in November 2016, suggests severity of airflow limitation (GOLD 1 to 4) and assessment of symptoms and exacerbation history (Group A to D) should be considered separately to make treatment more precisely [3]. Reducing exacerbation is the most essential goal in this study, so COPD with higher risk of exacerbation (in group B, C, D) will be enrolled.

Long-acting muscarinic antagonist (LAMA) and LABA are highly recommended by GOLD for patient in group B, C and D [22]. Although long-term inhalation of ICS is a high risk of pneumonia, there is no consensus on withdrawal of ICS from COPD treatment. The COSMIC study reported that withdrawal of fluticasone from combined salmeterol/fluticasone treatment aggravated decline of lung function, increased dyspnea and increased exacerbations in moderate and severe COPD patients [23]. In severe COPD patients with receiving tiotropium plus salmeterol/fluticasone, long-term withdrawal of fluticasone showed a significant decline in lung function [24]. Compared with salmeterol/fluticasone, recent researches showed that budesonide/formoterol had lower risk of adverse events [19]. So, for the sake of decreasing the effects of different inhaled drugs, basic treatment of each participant will be selected from tiotropium (18μg once q.d), budesonide/formoterol (160μg/4.5μg once or twice b.i.d) or combination of tiotropium(18μg) once daily and budesonide/formoterol (160μg/4.5μg once or twice b.i.d) in our study.

Budesonide/formoterol is believed to be an ideal salvational intervention drug. Firstly, budesonide/formoterol shows rapid-acting effects in COPD. Budesonide/formoterol significantly increases morning peak expiratory flow (PEF) and FEV_1_ in 5 min after inhalation, and increases morning activities including getting washed, dried, dressed, eating breakfast and walking around the home [17, 25]. Secondly, adding budesonide/formoterol to basic treatment might be safe. Tobias Welte et al. demonstrated that treatment of budesonide/formoterol (320μg/9μg) twice a day added to tiotropium not only improved lung function and symptoms of COPD patients but also highly reduced the morning, nighttime, and daytime use of terbutaline as reliever. Pharmacologically predictable adverse events related to treatment was rare and comparable across treatment groups [26]. In our study, the salvational intervention is basic treatment plus budesonide/formoterol (160μg/4.5μg once b.i.d) from the first day after severe air pollution exposure to the third day after air pollution end. For COPD patients treated with tiotropium (18μg once q.d) with or without budesonide/formoterol (160μg/4.5μg once b.i.d), the maximal dose of budesonide/formoterol per day during salvational intervention is no more than the dose of Tobias Welte’s research, indicating the salvational intervention should be safe for COPD. Conversely, for those COPD patients using budesonide/formoterol (160μg/4.5μg twice b.i.d) with or without tiotropium (18μg q.d), the tolerability of six-time usage of budesonide/formoterol (160μg/4.5μg) has not been reported in COPD yet. The tolerability will be concerned and determined in our study.

A limitation is that the study is not a double-blind design because both participants and researchers will know treatment which will be administered. To decrease the potential bias, investigators performing follow-up visits after randomization will be not informed of the administration of participants. Moreover, participants are asked to record daily dose of budesonide/formoterol (160μg/4.5μg) and investigators are asked to record daily AQI and whether the intervention is done.

Conclusion, this is a multi-center, prospective, randomized and standard treatment parallel control study aiming at reducing air pollution related COPD exacerbations. Evidence of the study will provide effectiveness and safety for a novel and precise strategy called the salvational intervention.

### Trail status

The trial is currently at the stage of patient recruitment and data collection.

## Abbreviations

AECOPD: Acute exacerbation of chronic obstructive pulmonary disease
AQI: Air quality index
CAT: COPD Assessment test
COPD: Chronic obstructive pulmonary disease
CT group: Control group
ICS: Inhaled corticosteroids
LABA: Long-acting beta2-agonists
LAMA: Long-acting muscarinic antagonist
mMRC: Modified british medical research council
PM: Particulate matter
SGRQ: St. George’s respiratory questionnaire
SI group: Salvational intervention group
